# Citrulline in the management of patients with urea cycle disorders

**DOI:** 10.1186/s13023-023-02800-8

**Published:** 2023-07-21

**Authors:** Apolline Imbard, Juliette Bouchereau, Jean-Baptiste Arnoux, Anaïs Brassier, Manuel Schiff, Claire-Marine Bérat, Clément Pontoizeau, Jean-François Benoist, Constant Josse, François Montestruc, Pascale de Lonlay

**Affiliations:** 1 Department of Biochemistry, Assistance Publique-Hôpitaux de Paris, Paris, France; 2 Université Paris-Saclay, Paris, France; 3 Reference Center for Inborn Error of Metabolism, Department of Pediatrics, Necker Hospital, Assistance Publique-Hôpitaux de Paris, G2M network, MetabERN, Paris, France; 4 Université de Paris, Paris, France; 5 Inserm UMR _S1163, Institut Imagine, Paris, France; 6 eXYSTAT, Malakoff, 92240 France; 7 Inserm UMR S1151, Institut Necker-Enfants Malades (INEM), Paris, France

**Keywords:** Ammonia, Arginine, Citrulline, Urea cycle disorders

## Abstract

**Background:**

Treatment recommendations for urea cycle disorders (UCDs) include supplementation with amino acids involved in the urea cycle (arginine and/or citrulline, depending on the enzyme deficiency), to maximize ammonia excretion through the urea cycle, but limited data are available regarding the use of citrulline. This study retrospectively reviewed clinical and biological data from patients with UCDs treated with citrulline and/or arginine at a reference center since 1990. The aim was to describe the prescription, impact, and safety of these therapies. Data collection included patient background, treatment details, changes in biochemical parameters (plasma ammonia and amino acids concentrations), decompensations, and patient outcomes.

**Results:**

Overall, 79 patients (median age at diagnosis, 0.9 months) received citrulline and/or arginine in combination with a restricted protein diet, most with ornithine transcarbamylase (n = 57, 73%) or carbamoyl phosphate synthetase 1 (n = 15, 19%) deficiencies. Most patients also received ammonium scavengers. Median follow-up was 9.5 years and median exposure to first treatment with arginine + citrulline, citrulline monotherapy, or arginine monotherapy was 5.5, 2.5, or 0.3 years, respectively. During follow-up, arginine or citrulline was administered at least once (as monotherapy or in combination) in the same proportion of patients (86.1%); the overall median duration of exposure was 5.9 years for arginine + citrulline, 3.1 years for citrulline monotherapy, and 0.6 years for arginine monotherapy. The most common switch was from monotherapy to combination therapy (41 of 75 switches, 54.7%). During treatment, mean ammonia concentrations were 35.9 µmol/L with citrulline, 49.8 µmol/L with arginine, and 53.0 µmol/L with arginine + citrulline. Mean plasma arginine concentrations increased significantly from the beginning to the end of citrulline treatment periods (from 67.6 µmol/L to 84.9 µmol/L, P < 0.05). At last evaluation, mean height and weight for age were normal and most patients showed normal or adapted behavior (98.7%) and normal social life (79.0%). Two patients (2.5%) experienced three treatment-related gastrointestinal adverse reactions.

**Conclusions:**

This study underlines the importance of citrulline supplementation, either alone or together with arginine, in the management of patients with UCDs. When a monotherapy is considered, citrulline would be the preferred option in terms of increasing plasma arginine concentrations.

**Supplementary Information:**

The online version contains supplementary material available at 10.1186/s13023-023-02800-8.

## Introduction

Urea cycle disorders (UCDs) are rare genetic disorders that result from deficiencies in enzymes or transporters of the urea cycle pathway [1, 2]. A large proportion of cases present with neonatal hyperammonemia, with a high risk of mortality or neurological impairment. Clinical presentation depends on whether the deficiency is partial or complete. Patients with partial deficiencies may have later onset and more variable clinical manifestations, but still have an increased risk of morbidity and mortality. As brain damage is directly related to the duration and severity of hyperammonemia, prompt diagnosis and treatment are imperative [2–4].

The long-term care of patients with UCDs involves normalizing ammonia concentrations and preventing decompensation by balancing nitrogen input and output. Therapeutic guidelines recommend a restriction of dietary protein, essential vitamins and minerals, ammonium scavengers (sodium benzoate and/or sodium phenylbutyrate) and supplementation with amino acids involved in the urea cycle (arginine and/or citrulline) [2, 5]. The number and the doses of treatments depend on the onset and severity of the disease.

UCDs caused by mitochondrial enzyme deficiencies induce an arginine deficiency, so patients with deficiencies in N-acetylglutamate synthase (NAGS), carbamoyl phosphate synthetase 1 (CPS1), ornithine transcarbamylase (OTC), or ornithine translocase (ORNT1) require supplementation with either arginine or its precursor, citrulline [2]. UCD treatment guidelines state that in patients with these mitochondrial deficiencies, supplementation with citrulline may be used instead of arginine; however, they also note that there have been no studies comparing both supplements [2]. By contrast, UCDs caused by cytosolic enzyme deficiencies are treated differently; citrulline is not recommended for individuals with argininosuccinate lyase deficiency and citrullinemia, and supplementation with arginine is not recommended for arginase 1 deficiency [2].

While supplementation with arginine and/or citrulline has an established role in treating UCDs, there is little data on treatment patterns or patient outcomes. Two retrospective studies have provided insights into using citrulline in patients with OTC [6, 7] or CPS1 [7]. The current study aimed at expanding this research by retrospectively collecting data for patients with CPS1, OTC, ORNT1, or NAGS deficiencies who received supplementation with arginine and/or citrulline. The aim was to investigate the use of citrulline, either alone or in combination with arginine, with a focus on the prescription, impact, and safety of such supplementation.

## Methods

### Study design and patient population

This retrospective study used medical data from patients with a UCD due to a defect in NAGS, CPS1, OTC, or ORNT1, who had been exposed to citrulline and/or arginine since 1990 at the Reference Center for Inherited Metabolic Disorders, Necker University Hospital – Enfants Malades, in Paris, France. Standard treatment practices for UCDs have not undergone major changes since 1990, even after new guidelines [1, 2] were implemented in 2012. Amino acids (citrulline and arginine) and ammonium scavengers have commonly been prescribed, with doses dependent on the severity of the disease, together with a protein restriction diet. Data from newborn patients (up to 1 month of age) who did not survive the initial episode of hyperammonemia were excluded. This research was conducted according to the principles of the Declaration of Helsinki and local and international laws. As per regulations, this study required patient non-objection. A written note informing the patient and/or their legal guardians/parents of the use of their data, along with their right to refuse this use, was sent by post or given during a regular consultation. If the patient was deceased, the investigator ensured that they had not, prior to their death, objected in writing to the use of their personal data. This research was registered on the French National Institute for Health Data directory (Institut National Des Données de Santé [INDS]) under the number MR 2,514,090,818.

### Evaluation criteria

The evaluation criteria included: treatment with citrulline and/or arginine and other treatments prescribed for UCD; any changes in biochemical parameters (plasma ammonia and fasting concentrations of citrulline, arginine, and glutamine); demographic and clinical characteristics of the population, including the height and weight, and mental, behavioral, and social development of patients at diagnosis and during treatment; the number of decompensations after the start of treatment (not including the diagnosis episode) and causes of decompensation; and the tolerability of citrulline and arginine. A decompensation episode was defined by either a decompensation visit being recorded in the patient’s medical records, or an episode identified by the patient having more than two ammonia values above the predefined critical threshold over two consecutive days combined with a change in their therapeutic management (modification of ammonium scavengers). More than two successive ammonia values between 50 and 100 µmol/L (normal range < 50 µmol/L), within two consecutive days was defined as a minor decompensation episode, and more than two successive ammonia values > 100 µmol/L within two consecutive days was defined as a major decompensation episode. Citrulline, arginine, and glutamine concentrations were compared with the standard reference concentrations used at the study center.

A treatment period was defined as the time interval during which a patient received either citrulline monotherapy, arginine monotherapy, or arginine + citrulline, with no change in drug regimen (i.e., stopping or (re)introducing citrulline or arginine).

Adverse drug reactions were defined as those occurring or worsening after treatment initiation and up to 30 days after treatment discontinuation.

### Statistical analyses

The exposed population included patients who were receiving or had received oral treatment with citrulline and/or arginine for their UCD and had at least one assessment after initiation of this treatment. All analyses were performed on the exposed population, except the analysis of decompensation, which was performed on the evaluable population.

The evaluable population included patients from the exposed population who, based on the investigator’s judgement through analysis of the patient files, had good treatment compliance and did not present with several episodes of decompensation despite appropriate medical care, as these factors could have interfered with the study evaluation. This population was validated during a data review meeting before database lock.

For some relevant data, results were displayed to show distribution between neonatal (revealed before 1 month old) and late onset (revealed after 1 month old) form of the disease.

The statistical analyses were performed by eXYSTAT (Malakoff, France) using SAS software version 9.4 (SAS Institute, North Carolina, USA). Descriptive statistics were used with continuous variables presented as number (n), number of missing data, mean ± standard deviation (SD), and median with minimum and maximum, and discrete variables presented as n, missing n, frequency, and percentages. Comparisons of the quantitative data were made using analysis of variance (ANOVA) or the non-parametric Kruskal Wallis test. Qualitative data comparisons were made using the chi-squared test or Fisher’s exact test based on population.

## Results

### Patient disposition and characteristics

A total of 86 potential study participants had UCDs related to deficiencies of OTC, CPS1, NAGS or ORNT1 and were followed by the reference center between January 1st 1990 and January 14th 2020. Of these, six patients (4 females and 2 males) were excluded because they did not receive arginine or citrulline and another was excluded because he could not be contacted and informed about the collection of his data. Seventy-nine patients (91.9%) satisfied the selection criteria and comprised the exposed population (Supplementary Fig. 1; Additional File 1). Follow-up duration in this exposed population ranged from 0.2 to 36.4 years (median 9.5 years; mean ± SD 11.2 ± 8.5 years; Supplementary Table 1; Additional File 2).

The median age at diagnosis (exposed population) was 0.9 months (range: 0.0 months – 46.0 years, Table 1).

**Table 1:** Patient characteristics/biochemical parameters at diagnosis, in total and according to enzyme deficiency (exposed population)

	OTC (n = 57)	CPS1 (n = 15)	NAGS (n = 4)	ORNT1 (n = 3)	Total (n = 79)
Female, n (%)	35 (61.4)	5 (33.3)	2 (50.0)	2 (66.7)	44 (55.7)
Male, n (%)	22 (38.6)	10 (66.7)	2 (50.0)	1 (33.3)	35 (44.3)
Age, years, median (range)	1.2 (0.0–46.0)	0.0 (0.0–37.2)	0.0 (0.0–0.1)	8.6 (0.0–9.0)	0.9 (0.0–46.0)
Neonatal presentation (diagnosed 0–30 days after birth), n (%)	16 (28.1)	10 (66.7)	4 (100.0)	1 (33.3)	31 (39.2)
Late-onset presentation (diagnosed 1 month after birth), n (%)	41 (71.9)	5 (33.3)	0	2 (66.7)	48 (60.8)
Diagnosis after a decompensation episode, n (%)	36 (63.2)	14 (93.3)	3 (75.0)	0	53 (67.1)
Diagnosis following risk assessment in relatives/family members	9 (15.8%)	1 (6.7%)	1 (25.0%)	1 (33.3%)	12 (15.2)
Diagnosis in patients with symptoms suggestive of UCD (out of decompensation)	12 (21%)	0 (0.0%)	0 (0.0%)	2 (66.6%)	14 (17.7)
Family history of UCD, n (%)	22 (38.6)	3 (20.0)	1 (25.0)	0 (0.0)	26 (32.9)
Family consanguinity, n (%)	2 (3.5)	4 (26.7)	4 (100.0)	1 (33.3)	11 (13.9)
Biochemical parameters, median (range)					
Plasma concentrations					
Ammonia, µmol/L	95.5 (28.5–1180.0) [n = 40]	123.5 (57.5–700.0) [n = 14]	64.0 (58.0–392.0) [n = 3]	217.5 (104.0–331.0) [n = 2]	104.0 (28.5–1180.0) [n = 59]
Citrulline, µmol/L	13.0 (2.0–147.0) [n = 34]	4.5 (0.0–39.0) [n = 13]	2.8 (0.0–5.5) [n = 2]	52.0 (52.0–52.0) [n = 1]	10.5 (0.0–147.0) [n = 50]
Arginine, µmol/L	33.0 (5.0–166.0) [n = 35]	32.0 (14.5–109.0) [n = 12]	124.5 (40.0–209.0) [n = 2]	87.0 (87.0–87.0) [n = 1]	34.0 (5.0–209.0) [n = 50]
Glutamine, µmol/L	873.8 (458.0–3941.5) [n = 38]	958.0 (472.0 − 2000.0) [n = 14]	1301.0 (695.0 − 1907.0) [n = 2]	1311.0 (1311.0–1311.0) [n = 1]	931.0 (458.0–3941.5) [n = 55]
Valine, µmol/L	120.0 (53.0–236.0) [n = 30]	95.5 (67.0–221.0) [n = 13]	133.5 (129.0–138.0) [n = 2]	197.0 (197.0–197.0) [n = 1]	118.0 (53.0–236.0) [n = 46]
Isoleucine, µmol/L	36.0 (9.0–61.0) [n = 31]	26.0 (13.0–74.0) [n = 13]	43.0 (39.0–47.0) [n = 2]	68.0 (68.0–68.0) [n = 1]	36.0 (9.0–74.0) [n = 47]
Urine orotic acid, mmol/mol/creatinine	8.4 (1.9–1381.0) [n = 18]	1.8 (1.8–1.8) [n = 1]	2.5 (2.5–2.5) [n = 1]	473.0 (473.0–473.0) [n = 1]	5.8 (1.8–1381.0) [n = 21]

CPS1, carbamoyl phosphate synthetase 1; NAGS, N-acetylglutamate synthase; ORNT1, ornithine translocase; OTC, ornithine transcarbamylase; UCD, urea cycle disorders.

The majority of patients in the exposed population had an OTC deficiency (72.2%; 35 females (7 with neonatal and 28 with late onset presentation), 22 males (9 with neonatal and 13 with late onset presentation); 19% (5 females, 10 males) had a CPS1 deficiency, and only small numbers had a NAGS (5.1%; 2 females, 2 males) or ORNT1 (3.8%; 2 females, 1 male) deficiency. Family consanguinity was reported in 11 patients overall (13.9%; in 2 patients with OTC Deficiency, 4 patients with CPS1 Deficiency, 4 patients with NAGS Deficiency and 1 patient with ORNT1 Deficiency) (Table 1).

Thirty one patients (39.2%) had a neonatal presentation (16 OTCD, 10 CPS1D, 4 NAGSD and 1 ORNT1D), and 48 (60.8%) patients had a late-onset form of the disease that was revealed after 1 month old (41 OTCD, 5 CPS1D and 2 ORNT1D).

UCD was diagnosed after a decompensation episode in 53 patients (67.1%), during risk assessment with attention to other relatives/family members in 12 patients (15.2%) or following biological metabolic work-up in patients with symptoms suggestive of UCD (n = 14, 17.7%).

Most patients were symptomatic at diagnosis (87.5%), with heterogeneous presentation between patients. The most common symptoms at diagnosis were neurological (87.5% of symptomatic patients), followed by digestive and hepatic symptoms (73.2%). Plasma ammonia concentrations at diagnosis were available in 59 patients, and ranged from 28.5 to 1180.0 µmol/L (median 104.0 µmol/L; normal < 100 µmol/L for newborns or < 50 µmol/L for others). Glutamine concentrations at diagnosis (n = 55) ranged from 458.0 to 3941.5 µmol/L (median 931.0 µmol/L; normal 530 ± 81 µmol/L; Table 1). The median concentration of plasma glutamine at baseline per deficiency were: 1311.0 µmol/L in the patient with ORNT1 deficiency; 1301.0 µmol/L (695.0–1907.0) in the two patients with NAGS deficiency; 958.0 µmol/L (472.0–2000.0) in the 14 patients with CPS1 deficiency and 873.8 µmol/L (458.0–3941.5) in the 38 patients with OTC deficiency.

Median plasma concentrations were low for citrulline (10.5 µmol/L, normal 26 ± 8 µmol/L) and arginine (34.0 µmol/L, normal 79 ± 25 µmol/L).

Patients with a neonatal presentation had increased mortality (12.9% in neonatal onset, whereas no death was registered in patients with late onset), peak plasma ammonium (mean value(SD): 220.1 (278.4) µmol/L versus 144.7 (113.0)µmol/L) and glutamine (mean value(SD): 1219.7 (885.5)µmol/L versus 955.5 (344.4) µmol/L) concentrations at diagnosis compared to late onset presentation.

### Arginine and/or citrulline treatment

During the first period after diagnosis, the most common initial treatment in the exposed population was oral arginine monotherapy (58.2% of patients; 54.8% in neonatal onset versus 60.4% in late-onset), followed by oral arginine + citrulline (24.1% of patients; 25.8% in neonatal onset versus 22.9% in late-onset) and oral citrulline monotherapy (17.7% of patients; 19.4% in neonatal onset versus 16.7% in late-onset, Table 2). These treatments replaced initial intravenous arginine, which was administered in most patients before diagnosis when UCD was suspected, and were administered, according to the guidelines [1, 2], in combination with other therapies, including emergency treatment with high-caloric glucose and lipids, reduced protein intake, and ammonium scavengers, depending on the severity of the UCD.

**Table 2:** Initial treatment with arginine and/or citrulline after diagnosis and during follow up (exposed population)

	OTC (n = 57)	CPS1 (n = 15)	NAGS (n = 4)	ORNT1 (n = 3)	Total (n = 79)
Initial treatment after diagnosis, n (%)					
Arginine	35 (61.4)	6 (40.0)	3 (75.0)	2 (66.7)	46 (58.2)
Citrulline	11 (19.3)	1 (6.7)	1 (25.0)	1 (33.3)	14 (17.7)
Arginine + citrulline	11 (19.3)	8 (53.3)	0	0	19 (24.1)
Duration of exposure to initial treatment, years, median (range)					
Arginine and/or citrulline	0.7 (0.0–23.9)	0.9 (0.0–15.4)	0.8 (0.0–6.9)	2.5 (1.9–9.1)	0.9 (0.0–23.9)
Arginine	0.3 (0.0–23.9)	0.2 (0.0–6.9)	0.2 (0.0–1.4)	5.5 (1.9–9.1)	0.3 (0.0–23.9)
Citrulline	2.5 (0.0–19.2)	0.6 (0.6–0.6)	6.9 (6.9–6.9)	2.5 (2.5–2.5)	2.5 (0.0–19.2)
Arginine + citrulline	3.6 (0.1–18.8)	8.9 (0.2–15.4)	–	–	5.5 (0.1–18.8)
Received any time during follow up, n (%)					
Arginine (± citrulline)	48 (84.2)	15 (100)	3 (75.0)	2 (66.7)	68 (86.1)
Citrulline (± arginine)	49 (86.0)	14 (93.3)	3 (75.0)	2 (66.7)	68 (86.1)
Received only arginine during follow up, n (%)	8 (14.0)	1 (6.7)	1 (25.0)	1 (33.3)	11 (13.9)
Received only citrulline during follow up, n (%)	9 (15.8)	0	1 (25.0)	1 (33.3)	11 (13.9)
Received only arginine + citrulline during follow up, n (%)	4 (7.0)	6 (40.0)	0	0	10 (12.7)
Received only arginine at least once during follow up, n (%)	38 (66.7)	6 (40.0)	3 (75.0)	2 (66.7)	49 (62.0)
Received only citrulline at least once during follow up, n (%)	22 (38.6)	5 (33.3)	2 (50.0)	1 (33.3)	30 (38.0)
Received arginine + citrulline at least once during follow up, n (%)	38 (66.7)	13 (86.7)	2 (50.0)	1 (33.3)	54 (68.4)
Time from diagnosis to first treatment with arginine and/or citrulline, years					
Arginine					
Mean (SD)	1.9 (5.3)	0.1 (0.1)	0.1 (0.2)	0.1 (0.1)	1.5 (4.7)
Median (range)	0.0 (0.0–25.9)	0.0 (0.0–0.3)	0.0 (0.0–0.3)	0.1 (0.0–0.2)	0.0 (0.0–25.9)
Citrulline					
Mean (SD)	2.9 (3.5)	4.1 (6.6)	1.4 (1.0)	0.1 (NA)	2.9 (3.9)
Median (range)	1.4 (0.0–11.5)	0.4 (0.4–15.6)	1.4 (0.7–2.2)	0.1 (0.1–0.1)	1.2 (0.0–15.6)
Arginine + citrulline					
Mean (SD)	2.4 (5.4)	0.2 (0.4)	0.1 (0.1)	9.2 (NA)	1.9 (4.7)
Median (range)	0.1 (0.0–25.6)	0.0 (-0.1–1.1)^a^	0.1 (0.0–0.2)	9.2 (9.2–9.2)	0.0 (-0.1–25.6)
Duration of exposure, years, median (range)					
Arginine and/or citrulline	9.4 (0.5–25.7)	9.1 (0.2–28.4)	3.0 (1.4–6.9)	2.5 (1.9–35.2)	7.3 (0.2–35.2)
Arginine monotherapy	0.6 (0.0–23.9)	0.2 (0.0–6.9)	1.4 (0.2–2.7)	5.5 (1.9–9.1)	0.6 (0.0–23.9)
Citrulline monotherapy	3.7 (0.0–19.2)	3.2 (2.0–12.0)	3.5 (0.0–6.9)	2.5 (2.5–2.5)	3.1 (0.0–19.2)
Arginine + citrulline	5.2 (0.1–22.9)	9.5 (0.2–15.7)	1.5 (1.1–2.0)	26.2 (26.2–26.2)	5.9 (0.1–26.2)
Duration of exposure as a percentage of total follow up duration, median (range)					
Arginine and/or citrulline	99.8 (7.4–100.0)	99.8 (82.8–100.0)	57.8 (26.6–99.9)	98.7 (96.8–99.6)	99.8 (7.4–100.0)
Arginine monotherapy	6.2 (0.0–100.0)	2.1 (0.1–96.1)	72.3 (1.9–81.6)	62.2 (25.6–98.7)	7.4 (0.0–100.0)
Citrulline monotherapy	59.6 (0.2–100.0)	41.9 (17.0–94.0)	17.1 (0.3–33.9)	96.8 (96.8–96.8)	55.7 (0.2–100.0)
Arginine + citrulline	94.3 (0.5–100.0)	99.1 (6.0–100.0)	26.0 (24.5–27.6)	73.9 (73.9–73.9)	94.3 (0.5–100.0)

^a^One patient was treated for suspected UCD before diagnosis was confirmed

CPS1, carbamoyl phosphate synthetase 1; NAGS, N-acetylglutamate synthase; NA, not applicable; ORNT1, ornithine translocase; OTC, ornithine transcarbamylase; SD, standard deviation; UCD, urea cycle disorders

The mean delay between diagnosis and initiation of treatment was numerically shorter for arginine monotherapy (1.5 years) or arginine + citrulline (1.9 years) than for citrulline monotherapy (2.9 years). The median duration of exposure to arginine + citrulline or citrulline monotherapy as first treatment was numerically longer than for arginine monotherapy (median durations in total exposed population: 5.5 and 2.5 vs. 0.3 years, respectively (in neonatal form: 3.9, 2.3 vs. 0.0 years and in late-onset form: 9.5, 3.4 vs. 0.5 years, respectively)).

During the overall study period, 68 patients (86.1%) received arginine and 68 (86.1%) received citrulline at least once (as monotherapy or in combination, Table 2). Most UCD patients (n = 54; 68.4%) also received arginine + citrulline at least once during follow-up, with the highest use of the combination in patients with OTC deficiency (38/57; 66.7%) or CPS1 deficiency (13/15; 86.7%). Arginine monotherapy was received at least once during the study by 49 patients (62.0%), and citrulline monotherapy by 30 patients (38.0%). The median of total treatment duration with arginine and/or citrulline was 7.3 years (range: 0.2–35.2) and represented 99.8% of the follow-up duration of patients. Median duration of exposure to treatment during follow-up was longest for arginine + citrulline (5.9 years; 4.0 years in neonatal form and 7.0 years in late-onset form), followed by citrulline monotherapy (3.1 years; 2.1 years in neonatal form and 4.3 years in late-onset form), and shortest for arginine monotherapy (0.6 years, 0.1 years in neonatal form and 0.9 years in late-onset form).

**Fig. 1:**
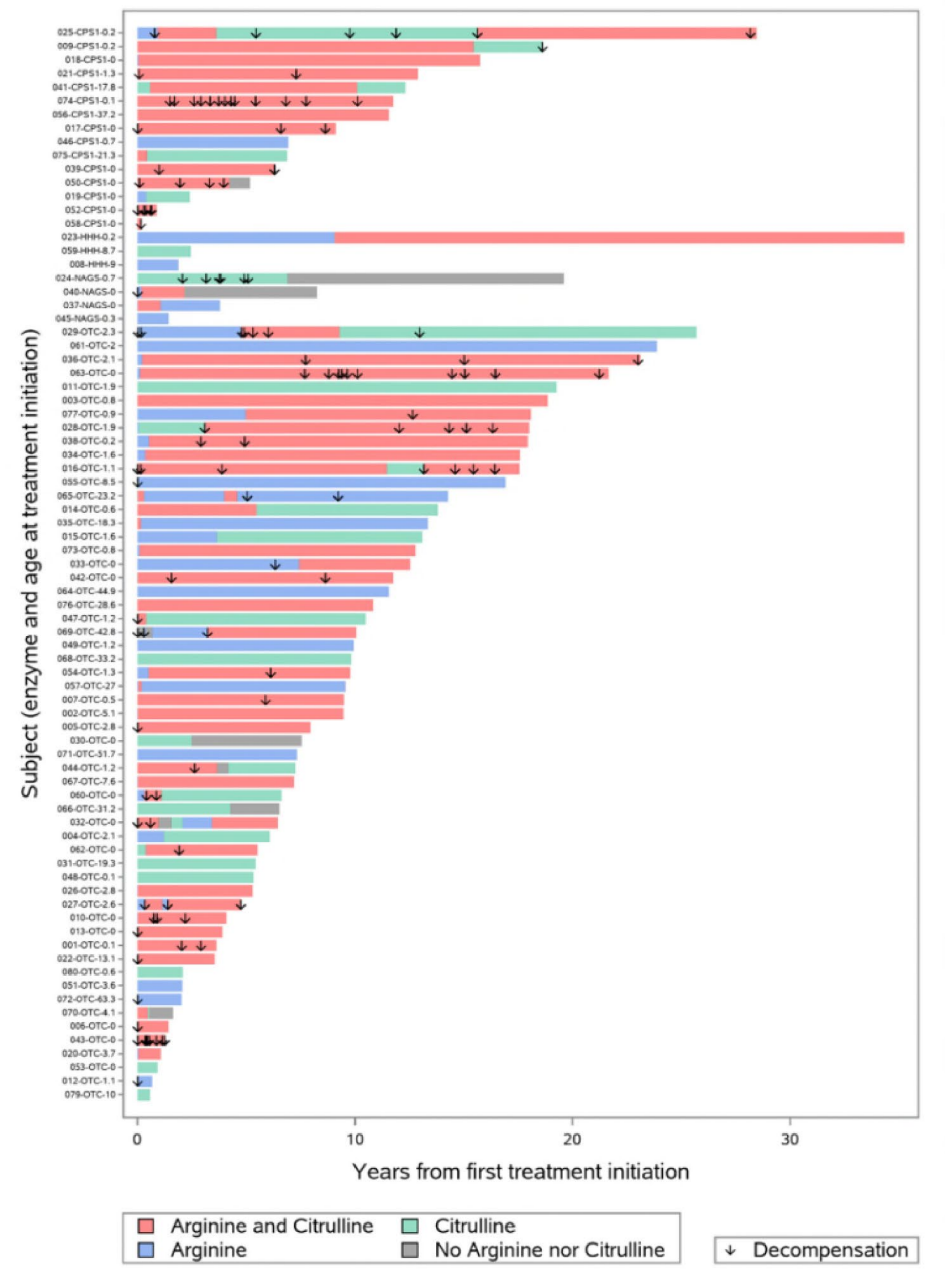
Global swimmer plot showing initial treatment for each patient after UCD diagnosis (exposed population, N = 79). CPS1, carbamoyl phosphate synthetase 1; HHH, hyperornithinemia-hyperammonemia-homocitrullinuria; NAGS, N-acetylglutamate synthase; OTC, ornithine transcarbamylase; UCD, urea cycle disorder

The 79 patients in the exposed population had a total of 147 treatment periods during follow-up: 60 with arginine + citrulline administration, 55 with arginine monotherapy, and 32 with citrulline monotherapy (Fig. 1; Table 3). The median duration of treatment periods was longest for arginine + citrulline (5.1 years), followed by citrulline monotherapy (3.1 years) and then arginine monotherapy (0.4 years, Supplementary Table 2; Additional File 2).

**Table 3:** Changes in treatment during follow up, as a proportion of treatment periods

	Arginine (n = 55)	Citrulline (n = 32)	Arginine + citrulline (n = 60)	Total (n = 147)
Treatment switched at end of treatment period^a^	41 (74.5)	13 (40.6)	21 (35.0)	75 (51.0)
Treatment(s) switched to:				
Arginine	–	2 (15.4)	6 (28.6)	8 (10.7)
Citrulline	4 (9.8)	–	11 (52.4)	15 (20.0)
Arginine + citrulline	36 (87.8)	5 (38.5)	–	41 (54.7)
No arginine or citrulline	1 (2.4)	6 (46.2)	4 (19.0)	11 (14.7)

Data are shown as number (%) of treatment periods

^a^Treatment periods were defined as a time interval receiving arginine alone, citrulline alone or arginine combined with citrulline, with no change in drug regimen (i.e., stopping or (re)introducing citrulline or arginine). There were a total of 147 treatment periods in the exposed population

**Fig. 2:**
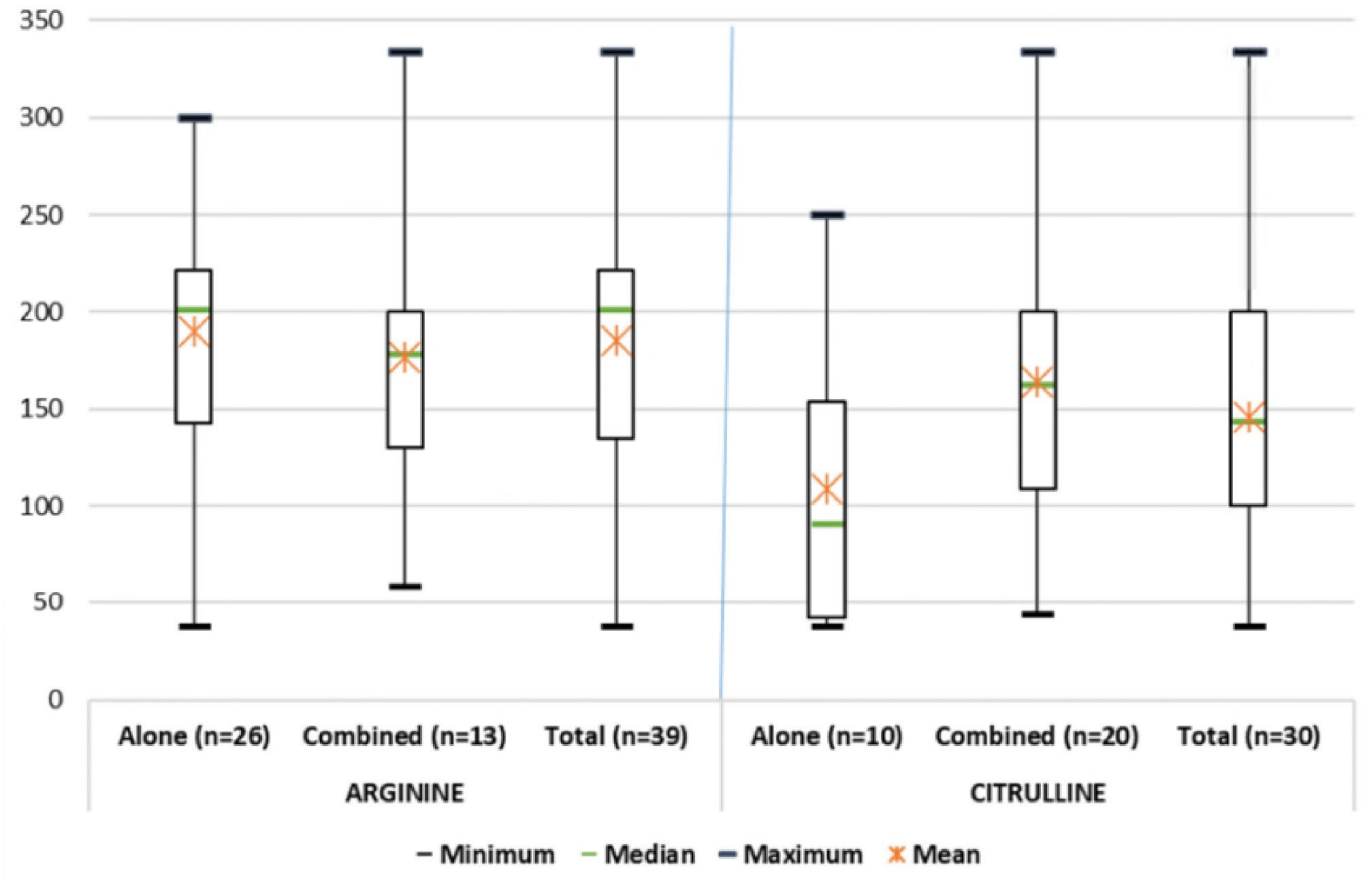
Arginine/citrulline doses (mg/kg/day), as monotherapy or combination therapy, at the beginning of a treatment period. Data were available for only 39 of the 115 treatment periods for arginine, and for 30 of the 92 treatment periods for citrulline (both as either monotherapy or part of combination therapy)

Where data were available, the median doses at the beginning of a treatment period with arginine and citrulline (either as monotherapy or as part of combination therapy) were 200.0 mg/kg/day and 143.2 mg/kg/day, respectively (Fig. 2). The daily dose was approximately 200 mg/kg/day for arginine (maximum dose 300 mg/kg/day) and approximately 150 mg/kg/day for citrulline (maximum dose 400 mg/kg/day) during the follow-up.

Among the 147 treatment periods, there were 75 (51.0%) treatment switches (Table 3). The most common switch was from monotherapy to combination therapy (41 of 75 switches, 54.7%). Switching treatment was more common after arginine monotherapy (74.5%) than after citrulline monotherapy (40.6%) or combined treatment (35.0%). After arginine monotherapy, citrulline was added in most cases (87.8%, n = 36). After citrulline monotherapy, treatment was switched to arginine + citrulline in 38.5% (n = 5) of cases, and to neither arginine nor citrulline in 46.2% of cases (n = 6).

Just over half of the 79 patients were receiving a combination treatment (51.9%; 61.3% in neonatal onset versus 45.8% in late-onset, Table 4) as the last supplementation given, 30.4% (32.3% in neonatal onset versus 29.3% in late-onset) were receiving citrulline monotherapy, and 17.7% arginine monotherapy (6.5% in neonatal onset versus 25% in late-onset). Overall, the median duration of exposure to final treatment with arginine and/or citrulline was 6.3 years (Table 4).

**Table 4:** Exposure to final treatment received during follow up period (exposed population)

	OTC (n = 57)	CPS1 (n = 15)	NAGS (n = 4)	ORNT1 (n = 3)	Total (N = 79)
Last treatment received, n (%)					
Arginine	10 (17.5)	1 (6.7)	2 (50.0)	1 (33.3)	14 (17.7)
Citrulline	17 (29.8)	4 (26.7)	2 (50.0)	1 (33.3)	24 (30.4)
Arginine + citrulline	30 (52.6)	10 (66.7)	0	1 (33.3)	41 (51.9)
Duration of exposure to last treatment, years					
Arginine and/or citrulline					
Mean (SD)	8.1 (6.3)	7.1 (5.0)	2.8 (3.0)	10.2 (13.9)	7.7 (6.3)
Median (range)	6.8 (0.0–23.9)	6.4 (0.2–15.7)	2.1 (0.0–6.9)	2.5 (1.9–26.1)	6.3 (0.0–26.1)
Arginine					
Mean (SD)	9.7 (7.2)	6.9 (NA)	2.1 (0.9)	1.9 (NA)	7.9 (6.9)
Median (range)	9.8 (0.7–23.9)	6.9 (6.9–6.9)	2.1 (1.4–2.7)	1.9 (1.9–1.9)	7.1 (0.7–23.9)
Citrulline					
Mean (SD)	6.3 (5.4)	3.5 (2.1)	3.4 (4.8)	2.5 (NA)	5.5 (4.9)
Median (range)	5.3 (0.1–19.2)	2.7 (2.0–6.4)	3.4 (0.0–6.9)	2.5 (2.5–2.5)	4.6 (0.0–19.2)
Arginine + citrulline					
Mean (SD)	8.5 (6.4)	8.5 (5.4)	NA	26.1 (NA)	9.0 (6.6)
Median (range)	7.0 (0.0–22.9)	10.3 (0.2–15.7)	NA	26.1 (26.1–26.1)	7.9 (0.0–26.1)

CPS1, carbamoyl phosphate synthetase 1; NAGS, N-acetylglutamate synthase; NA, not applicable; ORNT1, ornithine translocase; OTC, ornithine transcarbamylase; SD, standard deviation

In addition to arginine and/or citrulline supplementation, the most commonly administered other treatment for UCD was sodium benzoate, given in 86.4% of the 147 treatment periods; other UCD drugs administered were sodium phenylbutyrate (45.6%), carglumic acid (19.0%), and sodium phenylacetate/sodium benzoate (4.1%, Supplementary Table 3; Additional File 2). During all treatment periods, patients were prescribed a low-protein diet in addition to treatment. The median dose of natural proteins ranged between 0.0 (during decompensation) and 2.5 g/kg/day.

### Outcomes

#### Acute metabolic decompensation episodes

Acute metabolic decompensation episodes were analyzed during follow-up in the evaluable population only (n = 66, as 13/79 patients were excluded due to the lack of information regarding treatment intake), of whom 48 had an OTC deficiency, and 11, 4, and 3 patients had CPS1, NAGS, and ORNT1 deficiencies, respectively.

Overall, 36 patients (54.5%; 10 (35.7%) of neonatal patients and 26 (68.4%) of late-onset patients) exhibited no decompensation after the UCD diagnosis; the remaining 30 patients (18 of neonatal patients and 12 of late-onset patients) experienced a total of 98 acute metabolic decompensation episodes (excluding decompensations at diagnosis). Of these 30 patients, 10 (4 of neonatal patients and 6 of late-onset patients) had one decompensation episode, 7 had two decompensation episodes (5 of neonatal patients and 2 of late-onset patients), and 13 patients had more than two (9 of neonatal patients and 4 of late-onset patients). In patients whose initial decompensation episode was followed by another episode (n = 68 initial episodes), the median duration without decompensation was 0.9 years (range 0.0–12.5 years). Among all evaluable patients, the mean annual incidence of decompensation episodes was 0.20 during arginine + citrulline treatment (0.36 in neonatal and 0.09 in late onset), 0.04 during arginine monotherapy (0.08 in neonatal and 0.03 in late onset), and 0.10 during citrulline monotherapy (0.26 in neonatal and 0.03 in late onset). Respective annual incidences during these treatments were 0.18, 0.04, and 0.02 for patients with an OTC deficiency and 0.35, 0.12, and 0.29 for patients with a CPS1 deficiency.

Median plasma concentrations of ammonia reported during decompensation episodes (excluding decompensations at diagnosis) were 153.0 µmol/L in patients receiving arginine monotherapy, 100.5 µmol/L in patients receiving arginine + citrulline, 96.5 µmol/L in patients receiving citrulline monotherapy and 148.8 µmol/L in patients without any supplementation (Supplementary Table 4; Additional File 2). The ammonia concentration decreased markedly between the onset of decompensation and the next visit, in all treatment groups (mean [SD] change of − 67.5 [139.9] µmol/L).

The overall median duration of a decompensation episode was 5 days (range 1–82 days, Supplementary Table 4; Additional File 2). The duration of decompensation episodes in patients receiving arginine, combined supplementation, or no treatment were not statistically different from the duration in those receiving citrulline.

The median doses of arginine and citrulline remained broadly stable before and during UCD decompensations. All patients receiving citrulline and/or arginine at the onset of an episode also received at least one additional ammonia-scavenger during the decompensation episode.

#### Biochemical parameters during treatment periods

Basal plasma ammonia concentrations, i.e. not during decompensation episodes, decreased during all treatment periods (exposed population): The mean (SD) of the median ammonia concentrations during treatment periods were 35.9 (13.4) µmol/L during citrulline monotherapy, 49.8 (22.0) µmol/L during arginine monotherapy, and 53.0 (33.5) µmol/L during arginine + citrulline.

Mean plasma arginine concentrations increased significantly from the beginning to the end of treatment periods with citrulline (from 67.6 µmol/L to 84.9 µmol/L, P = 0.05), whereas increases were not significant with arginine (P = 0.76) or arginine + citrulline (P = 0.60; Table 5).

**Table 5:** Change in plasma arginine concentration from beginning to end of treatment periods

	Treatment		
	First value after start of treatment period	Last value available for treatment period	P-value^a^
During arginine treatment periods			
Plasma arginine concentration, µmol/L			
Mean (SD)	56.0 (35.1)	62.5 (49.8)	0.76
Median (Range)	46.0 (3.0–166.0)	49.0 (8.0–209.0)	
Data available (data missing), n	37 (18)	25 (30)	
During citrulline treatment periods			
Plasma arginine concentration, µmol/L			
Mean (SD)	67.6 (56.6)	84.9 (46.0)	0.05
Median (Range)	55.0 (10.0–318.0)	76.0 (39.0–231.0)	
Data available (data missing), n	25 (7)	19 (13)	
During arginine + citrulline treatment periods			
Plasma arginine concentration, µmol/L			
Mean (SD)	62.0 (43.4)	64.1 (38.6)	0.60
Median (Range)	52.0 (7.0–265.0)	58.0 (14.0–199.0)	
Data available (data missing), n	54 (6)	51 (9)	

Data shown are per treatment period (total 147 periods) in the exposed population (N = 79)

^a^Kruskal Wallis test

SD, standard deviation

Mean citrulline plasma concentrations remained low in all treatment periods. Mean (SD) concentrations were 23.2 (12.3) µmol/L during arginine treatment periods, 22.3 (17.0) µmol/L during arginine + citrulline periods, and 16.5 (8.1) µmol/L during citrulline periods.

There were reductions in plasma glutamine concentrations during treatment periods with arginine monotherapy (decreased from mean [SD] 1053.7 [690.0] to 700.5 [126.3] µmol/L) and citrulline monotherapy (mean [SD] 870.2 [273.4] to 719.2 [213.8] µmol/L). No significant change was perceived in patient with arginine + citrulline therapy (from mean [SD] 870.2 [435.5] to 917.0 [299.1] µmol/L).

#### Changes in patient characteristics, development, and social life

In the patients below 18 years of age, body weight, height, and head circumference were within normal ranges at birth and at first reference visits, as the median values of the z-scores for height, body weight, body mass index (BMI), and head circumference showed no growth delay (defined by a value less than 2 SDs according to growth charts). During follow-up, growth parameters remained broadly comparable to the general population, with mean and median z-scores indicating normal growth between diagnosis and their last medical follow-up performed before 18 years (Supplementary Fig. 2; Additional File 1).

As patients got older, they were more likely to be assessed for their development and social life (Supplementary Table 5; Additional File 2). At the final visit, of those evaluated, all but one patient showed normal or adapted behavior (75/76, 98.7%) and 49 of 62 (79.0%) had a normal social life.

#### Adverse drug reactions

During follow-up, two patients (2.5%) experienced a total of three arginine or citrulline-related adverse reactions, one while receiving arginine, and the other while receiving citrulline (Supplementary Table 6; Additional File 2). The patient receiving arginine had nausea. The patient receiving citrulline had abdominal pain and nausea, which led to treatment discontinuation.

## Discussion

This retrospective study analyzed data from 79 patients with UCDs who were treated with arginine and/or citrulline associated with nitrogen scavengers and followed up for between 0.2 and 36 years (median of 9.5 years; mean 11.2 years). To our knowledge, this is the first study to analyze longitudinal clinical data from one of the largest French reference centers managing patients with UCDs with a focus on the use of citrulline and/or arginine.

The most common initial supplementation after diagnosis was arginine monotherapy, followed by arginine + citrulline, and then citrulline monotherapy. The duration between diagnosis and initial treatment with arginine monotherapy was shorter than for citrulline monotherapy (mean 1.5 vs. 2.9 years). There are at least two possible reasons for this difference. Firstly, physicians have to wait for results of chromatography of amino acids (CAA) to rule out citrullinemia, arginase 1 or argininosuccinate lyase deficiency before citrulline supplementation can be initiated. Secondly, arginine is available in an intravenous form, whereas citrulline is given orally, which may impact treatment initiation in the emergency setting.

Our data indicate that, while arginine is the most commonly used initial treatment, exposure to arginine + citrulline (median 5.5 years) or citrulline monotherapy (2.5 years) as initial treatment was longer than that of arginine monotherapy (0.3 years). Overall, ~ 86% of patients received arginine or citrulline at least once during follow-up and 68% received arginine + citrulline at least once during follow-up. Our data complement previous retrospective research from the same institution, which evaluated 90 French patients with OTC deficiencies, and showed that 70% of patients with the neonatal form of UCD received supplementation with both arginine and citrulline, underlining that the most severe UCD required combination therapy [6]. The combination of arginine + citrulline was also the most common practice during treatment episodes in our study. Patients were more likely to switch treatment after arginine monotherapy than after citrulline monotherapy or combination treatment, and the most common switch from arginine monotherapy was to combination treatment. According to guidelines, before diagnosis when UCD is suspected, arginine (including intravenous in case of decompensation) is used as an initial treatment. Once the diagnosis established, arginine could be kept with addition of citrulline (in severe forms) or switch to citrulline (without arginine), as this supplementation is more suitable for increasing citrulline concentrations. The availability of arginine in liquid form (while citrulline is available only in a powder enclosed in capsules) is also taken into consideration.

Treatment duration with arginine and/or citrulline was 7.3 years (median), which correlated to 99.8% of the follow up duration, indicating that most patients received almost constant supplementation with arginine and/or citrulline as soon as diagnosis was established. Median total duration of exposure was longest for arginine + citrulline (5.9 years), followed by citrulline monotherapy (3.1 years) and then arginine monotherapy (0.6 years), depending on the severity of the disease.

Taken together, these data indicated that oral citrulline is commonly prescribed for maintenance therapy in a post-acute setting, usually in combination with arginine. Treatment adherence is an essential determinant of outcomes in patients with UCDs [8], and non-adherence to diet or medication is an important trigger for hyperammonemic episodes [9, 10]. It is encouraging that a US drug adherence survey by Shchelochkov and colleagues of 52 adult UCD patients and 114 caregivers of young UCD patients found that 95% of patients were either compliant or very compliant with citrulline supplementation [11]. Adherence tends to be higher with amino acid supplementation than with nitrogen scavengers [11], as these latter could be considered less palatable by patients.

In our study, more than half of the patients (54.5%) who were considered to be compliant with treatment had no acute metabolic decompensations after the diagnostic episode during follow-up. The overall annual incidence of decompensation episodes was higher in patients receiving combination treatment than arginine or citrulline monotherapy, which again most likely reflects greater disease severity in patients receiving the combination. High concentrations of plasma ammonia were reported during decompensation episodes, particularly in patients receiving arginine monotherapy compared with those receiving citrulline monotherapy or combination therapy.

A long-term outcome study has shown that hyperammonemia is significantly correlated with poor neurological outcomes [12]. In the current study, there was a reduction in plasma ammonia concentrations with all treatments after the initial decompensation, including nitrogen scavengers. Excluding the decompensation periods, they were in a normal range irrespective of arginine or citrulline treatment, or subnormal in the group treated by arginine and citrulline, again underlining the severity of UCD in this group. There was also a reduction in plasma glutamine concentrations, with all treatments, more pronounced with arginine supplementation due to the fact that arginine was administered immediately after the initial decompensation in most patients. As some patients were switch to citrulline lately, the plasma glutamine concentration at treatment initiation did not perfectly reflect the initial plasma glutamine concentration at diagnosis or during the first episode of decompensation.

Treatment with citrulline significantly increased the mean plasma concentrations of arginine to within the normal range whereas increases of arginine concentrations s with arginine alone or arginine + citrulline treatments were not significant; indeed, mean plasma concentrations at the end of these treatment periods remained below normal. Similarly, other pharmacokinetic studies in healthy volunteers, including elderly individuals, citrulline increased plasma arginine concentrations significantly more effectively than arginine supplementation alone [13, 14], possibly because citrulline is converted to arginine through the renal pathway of arginine synthesis and that arginine is reabsorbed [15]. An analysis of a European multicenter registry (which included data for 361 patients with UCDs) showed that patients with CPS1, OTC, or ORNT1 deficiencies who received citrulline supplementation had significantly higher plasma arginine concentrations s than those who received only arginine supplementation or no supplementation [16]. The reason for this is likely to be pharmacokinetic; arginine undergoes extensive metabolism in the intestine and liver, whereas citrulline does not undergo intestinal metabolism and is not taken up by the liver [17], resulting in greater bioavailability [14]. This makes exogenous citrulline administration a more effective way of augmenting arginine concentrations than arginine administration [14]. As it is a rare disease and the benefits of citrulline in clinical practice has been established for decades, it is unsurprising that there are few studies of citrulline supplementation in patients with UCDs. A Japanese retrospective study of 43 patients with OTC or CPSD1 deficiencies showed that citrulline treatment significantly reduced serum ammonia concentrations (from 81.1 µmol/L before treatment to 36.8 µmol/L, P < 0.05) [7]. Similar to the current study, plasma arginine concentrations were significantly higher after the initiation of citrulline treatment, even when arginine doses were reduced. The authors concluded that citrulline supplementation could be the standard treatment for patients with OTC or CPSD1 deficiencies [7].

Hyperammonemia in UCD can cause encephalopathy, of which the long-term effects range from mild cognitive impairment to more severe intellectual disability [4]. Treatment with arginine reduced attacks of hyperammonemia in boys with late-onset OTC deficiency and led to an increase in their growth [18]. Although no brain function testing was performed in this study, at the end of follow-up, almost all patients (99%) displayed normal or adapted behavior and ~ 80% had a normal social life. This is consistent with previous data from France among patients with OTC deficiency, which showed that most pediatric patients with neonatal onset had normal or adapted schooling, and the adult patients had good socio-professional integration [6]. In the same way, at the end of follow-up, mean and median BMI, height and weight for age were in normal ranges even though patients with UCD frequently show growth impairment [19].

In the current study, supplementation with arginine and/or citrulline was well tolerated, with only two patients experiencing a total of three treatment-related gastrointestinal adverse events. In the above-mentioned US drug adherence survey conducted by Shchelochkov and colleagues, citrulline was reported to be better tolerated than arginine supplementation [11]. Patients in our study were receiving citrulline at a median daily dose that was consistent with the recommended dose in Europe (100–200 mg/kg [1]), and a little lower than the recommended dose in the USA (170 mg/kg [7]).

Strengths of this study are that it was performed at a reference center with a large cohort of patients with UCDs in France, where management practices were consistent over time, and with data collection covering 30 years and including all eligible patients at that center. However, it was a single-center study, and was retrospective in design. As a result, some selection bias is inherent in the study, e.g., the selection of combination therapy for patients with more severe disease. Other therapies could influence ammonia and plasma amino acid results; Sodium Phenylbutyrate and Phenylacetate may have a direct impact on glutamine concentration, making their dosage important. Malnutrition would also influence the baseline levels of glutamine. Hence, It is difficult to compare the efficacy of arginine, citrulline and arginine + citrulline on decompensation episode numbers and on biochemical parameters without taking into account disease severity, other treatments (ammonium scavengers) and environmental context (e.g. number of infections, malnutrition). However, this study underlines the efficacy of citrulline, alone or associated with other treatments, in metabolic balance of mitochondrial UCD and its active role on plasma arginine concentrations s. Since we relied on patients’ medical records as the source of data, there were inevitable gaps in the data for some patients and we could not distinguish the effects on outcomes of UCD treatments other than citrulline and arginine.

In conclusion, although the current study is retrospective, it underlines the importance of citrulline supplementation, either alone or with arginine, in managing most patients with UCD. In this cohort, citrulline was preferred for the medical management of patients resulting in positive metabolic outcomes. Indeed, citrulline achieved a good metabolic balance in patients with severe UCDs when used in association with arginine, or alone in less severe UCDs, and reaching normal plasma arginine concentrations. The results also suggest that citrulline supplementation may be more effective than arginine supplementation at increasing plasma arginine concentrations.

## Electronic supplementary material

Below is the link to the electronic supplementary material.

Supplementary Material 1

Supplementary Material 2

Supplementary Material 3

Supplementary Material 4

Supplementary Material 5

## Data Availability

The authors confirm that the data supporting the findings of this study are available within the article and its supplementary material. In order to comply with its obligation of transparency, Biocodex made this research publicly available (on August 9, 2018) in the public directory of the French National Institute for Health Data (Institut National Des Données de Santé [INDS]) under the number MR 2,514,090,818.

## Abbreviations

ANOVA: analysis of variance
BMI: body mass index
CAA: chromatography of amino acids
CPS1: carbamoyl phosphate synthetase 1
INDS: Institut National Des Données de Santé
N: number
NAGS: N-acetylglutamate synthase
ORNT1: ornithine translocase
OTC: ornithine transcarbamylase
SD: standard deviation
UCD: urea cycle disorders
US(A): United States (of America)

## References

[1] Häberle J, Boddaert N, Burlina A, Chakrapani A, Dixon M, Huemer M (2012). Suggested guidelines for the diagnosis and management of urea cycle disorders. Orphanet J Rare Dis.

[2] Häberle J, Burlina A, Chakrapani A, Dixon M, Karall D, Lindner M (2019). Suggested guidelines for the diagnosis and management of urea cycle disorders: first revision. J Inherit Metab Dis.

[3] Sen K, Anderson AA, Whitehead MT, Gropman AL (2021). Review of multi-modal imaging in urea cycle disorders: the old, the new, the borrowed, and the blue. Front Neurol.

[4] Sen K, Whitehead M, Castillo Pinto C, Caldovic L, Gropman A (2022). Fifteen years of urea cycle disorders brain research: looking back, looking forward. Anal Biochem.

[5] Filière de santé Maladies Rares G2M. Protocole National de Diagnostic et de Soins (PNDS): Déficits du cycle de l’urée. 2021. Available from: https://www.has-sante.fr/upload/docs/application/pdf/2021-06/pnds_ucd_vf.pdf Accessed 2 June 2022.

[6] Brassier A, Gobin S, Arnoux JB, Valayannopoulos V, Habarou F, Kossorotoff M (2015). Long-term outcomes in ornithine transcarbamylase deficiency: a series of 90 patients. Orphanet J Rare Dis.

[7] Tanaka K, Nakamura K, Matsumoto S, Kido J, Mitsubuchi H, Ohura T (2017). Citrulline for urea cycle disorders in Japan. Pediatr Int.

[8] Enns GM, Porter MH, Francis-Sedlak M, Burdett A, Vockley J (2019). Perspectives on urea cycle disorder management: results of a clinician survey. Mol Genet Metab.

[9] McGuire PJ, Lee HS, Summar ML, members of the Urea Cycle Disorders C (2013). Infectious precipitants of acute hyperammonemia are associated with indicators of increased morbidity in patients with urea cycle disorders. J Pediatr.

[10] Summar ML, Dobbelaere D, Brusilow S, Lee B (2008). Diagnosis, symptoms, frequency and mortality of 260 patients with urea cycle disorders from a 21-year, multicentre study of acute hyperammonaemic episodes. Acta Paediatr.

[11] Shchelochkov OA, Dickinson K, Scharschmidt BF, Lee B, Marino M, Le Mons C (2016). Barriers to drug adherence in the treatment of urea cycle disorders: assessment of patient, caregiver and provider perspectives. Mol Genet Metab Rep.

[12] Kido J, Matsumoto S, Häberle J, Nakajima Y, Wada Y, Mochizuki N (2021). Long-term outcome of urea cycle disorders: report from a nationwide study in Japan. J Inherit Metab Dis.

[13] Schwedhelm E, Maas R, Freese R, Jung D, Lukacs Z, Jambrecina A (2008). Pharmacokinetic and pharmacodynamic properties of oral L-citrulline and L-arginine: impact on nitric oxide metabolism. Br J Clin Pharmacol.

[14] Moinard C, Maccario J, Walrand S, Lasserre V, Marc J, Boirie Y (2016). Arginine behaviour after arginine or citrulline administration in older subjects. Br J Nutr.

[15] Brosnan ME, Brosnan JT (2004). Renal arginine metabolism. J Nutr.

[16] Molema F, Gleich F, Burgard P, van der Ploeg AT, Summar ML, Chapman KA (2019). Evaluation of dietary treatment and amino acid supplementation in organic acidurias and urea-cycle disorders: on the basis of information from a european multicenter registry. J Inherit Metab Dis.

[17] Cynober L (2007). Pharmacokinetics of arginine and related amino acids. J Nutr.

[18] Nagasaka H, Yorifuji T, Murayama K, Kubota M, Kurokawa K, Murakami T (2006). Effects of arginine treatment on nutrition, growth and urea cycle function in seven japanese boys with late-onset ornithine transcarbamylase deficiency. Eur J Pediatr.

[19] Kido J, Matsumoto S, Ito T, Hirose S, Fukui K, Kojima-Ishii K (2021). Physical, cognitive, and social status of patients with urea cycle disorders in Japan. Mol Genet Metab Rep.

