# Supplementary material for: Citrulline in the management of patients with urea cycle disorders
Source: Orphanet J Rare Dis. 2023 Jul 21;18:207. doi: 10.1186/s13023-023-02800-8 (PMC10362745; doi:10.1186/s13023-023-02800-8)

**Supplementary Figure 1.** **(a)** Patient flow. **(b)** Gender distribution

**(a)**


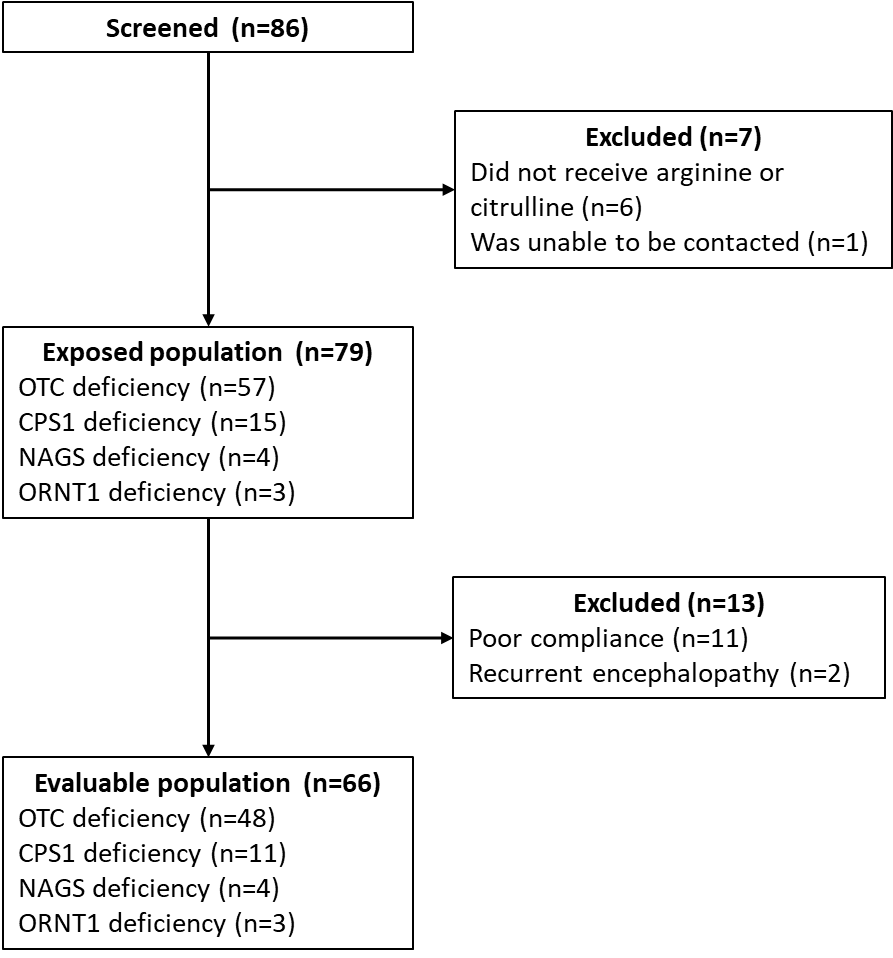


OTC, ornithine transcarbamylase; CPS1, carbamoyl phosphate synthetase 1; NAGS, N-acetylglutamate synthase; ORNT1, ornithine translocase.

**(b)**

|  | **Time of diagnosis** | | **Diagnosis > 1 month** | | | **Neo-Natal patients** | | | |  |
| --- | --- | --- | --- | --- | --- | --- | --- | --- | --- | --- |
|  | **>1 month (N=48)** | **Neo-Natal (N=31)** | **CPS1 (N=5)** | **HHH (N=2)** | **OTC (N=41)** | **CPS1 (N=10)** | **HHH (N=1)** | **NAGS (N=4)** | **OTC (N=16)** | **Total (N=79)** |
| Gender |  |  |  |  |  |  |  |  |  |  |
| n | 48 | 31 | 5 | 2 | 41 | 10 | 1 | 4 | 16 | 79 |
| Female | 31 (64.6%) | 13 (41.9%) | 1 (20.0%) | 2 (100%) | 28 (68.3%) | 4 (40.0%) | 0 (0.0%) | 2 (50.0%) | 7 (43.8%) | 44 (55.7%) |
| Male | 17 (35.4%) | 18 (58.1%) | 4 (80.0%) | 0 (0.0%) | 13 (31.7%) | 6 (60.0%) | 1 (100%) | 2 (50.0%) | 9 (56.3%) | 35 (44.3%) |
| Missing data | 0 | 0 | 0 | 0 | 0 | 0 | 0 | 0 | 0 | 0 |

**
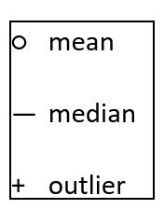
Supplementary Figure 2.** Change in body mass index (BMI) for age, height for age, and weight for age Z-scores from patient’s reference visit to final visit in patients aged up to 18 years: **(a)** total exposed population (n=79); **(b)** patients exposed to arginine (n=69); **(c)** patients exposed to citrulline (n=68).

**(a)**


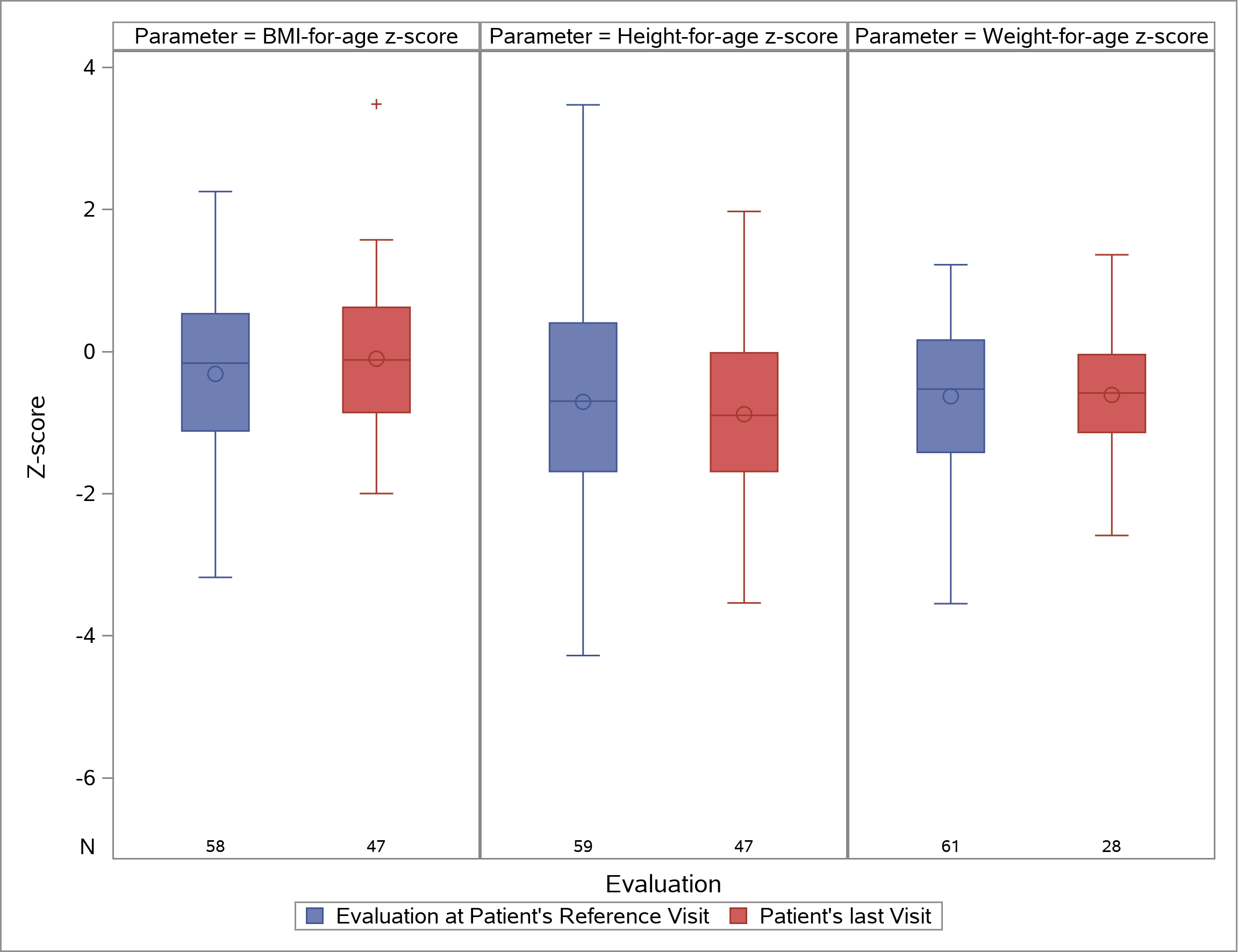


**(b)**


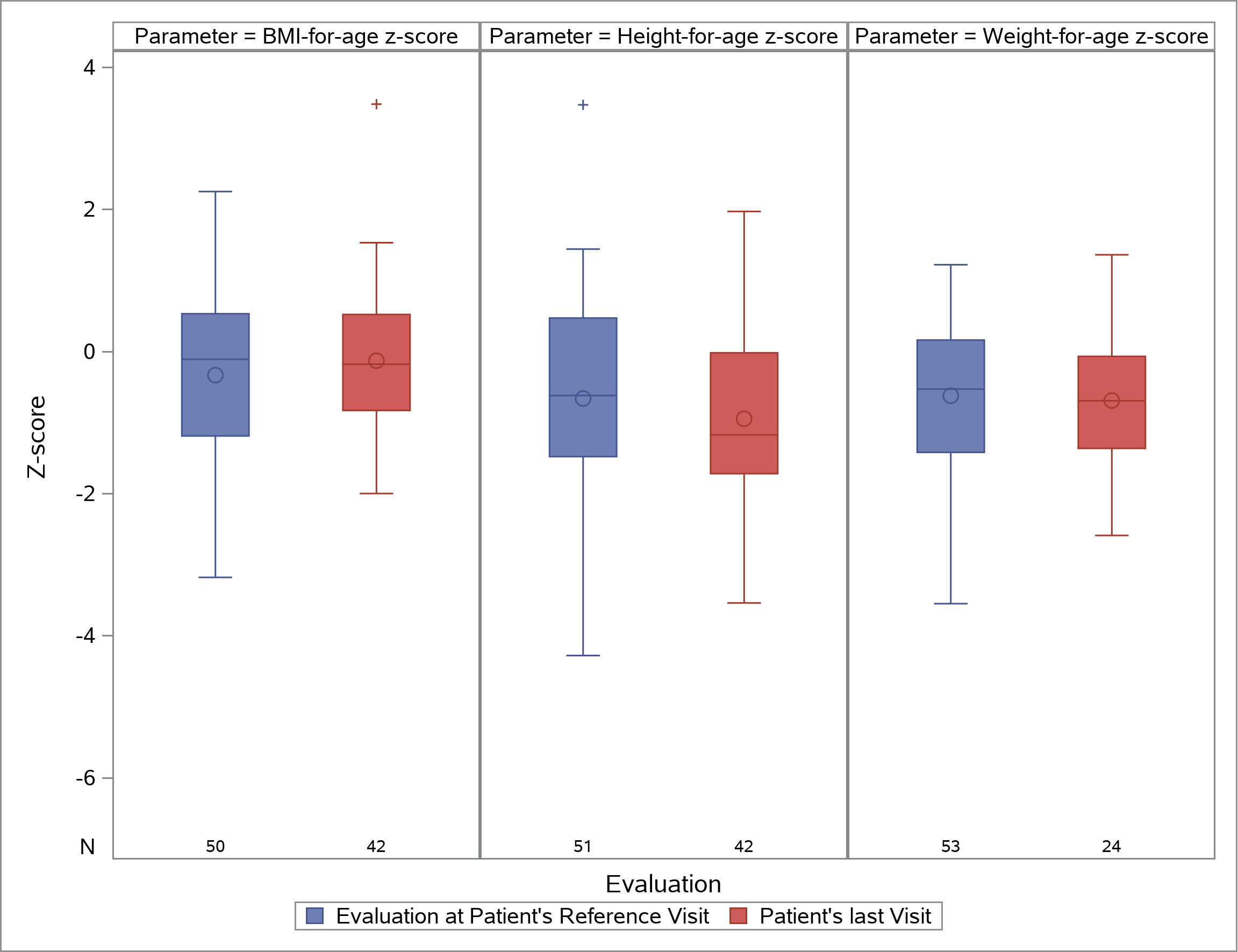


**(c)**


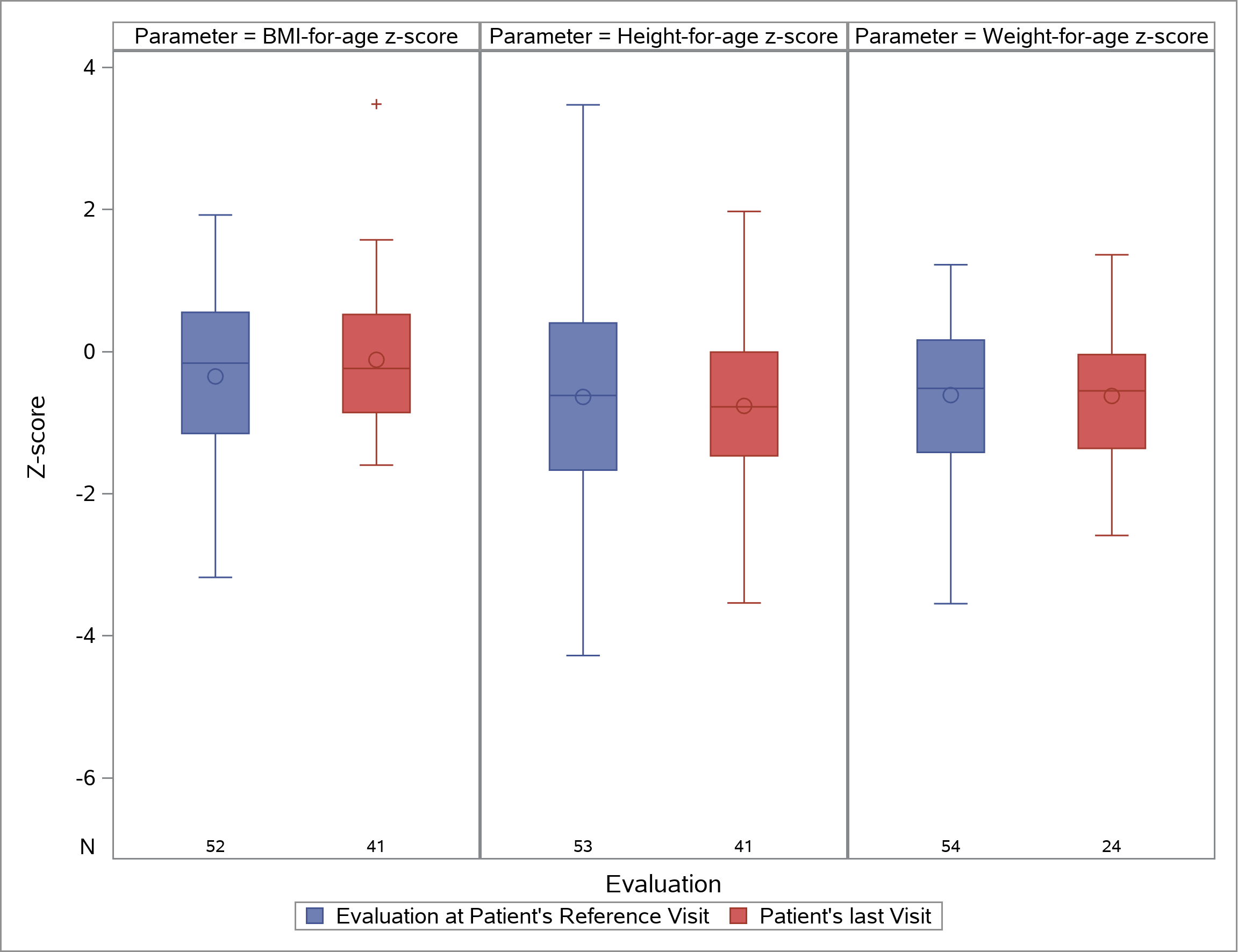

Supplement: Supplementary file 2 — Supplementary Material 2 [file 13023_2023_2800_MOESM2_ESM.docx]
