# Supplementary material for: Citrulline in the management of patients with urea cycle disorders
Source: Orphanet J Rare Dis. 2023 Jul 21;18:207. doi: 10.1186/s13023-023-02800-8 (PMC10362745; doi:10.1186/s13023-023-02800-8)
Supplement: Supplementary file 5 — Supplementary Material 5 [file 13023_2023_2800_MOESM5_ESM.docx]

**Supplementary Table 1.** Follow-up duration from diagnosis (exposed population), according to enzyme deficiency.

|  | **OTC (n=57)** | **CPS1 (n=15)** | **NAGS (n=4)** | **ORNT1 (n=3)** | **Total (n=79)** |
| --- | --- | --- | --- | --- | --- |
| Follow up duration, years from diagnosis | | | | | |
| Mean (SD) | 11.6 (8.2) | 10.0 (7.4) | 8.5 (8.3) | 13.3 (19.2) | 11.2 (8.5) |
| Median (range) | 9.9  (0.7–36.4) | 9.1  (0.2–28.6) | 6.0  (1.7–20.3) | 2.5  (1.9–35.4) | 9.5  (0.2–36.4) |

CPS1, carbamoyl phosphate synthetase 1; NAGS, N-acetylglutamate synthase; NA, not applicable; ORNT1, ornithine translocase; OTC, ornithine transcarbamylase; SD, standard deviation.

**Supplementary Table 2.** Duration of treatment period in years. Treatment periods were defined as a time interval receiving arginine alone, citrulline alone or arginine combined with citrulline, with no change in drug regimen (i.e., stopping or (re)introducing citrulline or arginine). There were a total of 147 treatment periods in the exposed population.

|  | **Arginine (n=55)** | **Citrulline (n=32)** | **Arginine + citrulline (n=60)** | **Total (n=147)** |
| --- | --- | --- | --- | --- |
| Duration of treatment period, years | | | | |
| Mean (SD) | 3.0 (4.9) | 4.7 (4.8) | 7.1 (6.5) | 5.0 (5.9) |
| Median (range) | 0.4 (0.0–23.9) | 3.1 (0.0–19.2) | 5.1 (0.0–26.1) | 2.7 (0.0–26.1) |

SD, standard deviation.

**Supplementary Table 3.** Other treatments received during treatment periods with arginine and/or citrulline. There were a total of 147 treatment periods in the exposed population.

|  | **Treatment period** | | | |
| --- | --- | --- | --- | --- |
|  | **Arginine (n=55)** | **Citrulline (n=32)** | **Arginine + citrulline (n=60)** | **Total  (n=147)** |
| Other treatments during treatment period*, n (%) | | |  |  |
| Sodium benzoate | 45 (81.8) | 23 (71.9) | 59 (98.3) | 127 (86.4) |
| Sodium phenylbutyrate | 18 (32.7) | 8 (25.0) | 41 (68.3) | 67 (45.6) |
| Carglumic acid | 11 (20.0) | 4 (12.5) | 13 (21.7) | 28 (19.0) |
| Sodium phenylacetate | 3 (5.5) | 0 | 3 (5.0) | 6 (4.1) |
| *Several other treatments could be given during the same treatment period | | | | |

**Supplementary Table 4. (a)** Description and duration of the 98 UCD decompensation episodes that occurred during follow up (excluding decompensation occurring at the diagnosis), and duration of periods without decompensations after a decompensation episode of the evaluable population; **(b)** Distribution and incidence of UCD Decompensation per treatment period.

**(a)**

|  | **Treatment at time of decompensation** | | | | **Total (n=98)** |
| --- | --- | --- | --- | --- | --- |
|  | **Arginine (n=4)** | **Citrulline (n=13)** | **Arginine and citrulline (n=77)** | **No arginine or citrulline** |  |
| Maximal plasma ammonia concentration reported during decompensation, µmol/L | | | | | |
| Mean (SD) | 393.8 (526.8) | 105.3 (46.6) | 111.1 (55.1) | 183.4 (104.6) | 126.0 (126.2) |
| Median (range) | 153.0 (89.0, 1180.0) | 96.5 (45.0, 225.0) | 100.5 (20.5, 285.0) | 148.8 (105.0, 331.0) | 100.8 (20.5, 1180.0) |
| Missing data, n | 0 | 0 | 8 | 0 | 8 |
| Change in plasma ammonia concentration from decompensation onset to the following visit, µmol/L | | | | | |
| Mean (SD) | –546.8 (816.4) | –19.2 (43.7) | –58.7 (62.4) | –79.4 (130.8) | –67.5 (139.9) |
| Median (range) | –546.8 (–1124.0, 30.5) | –13.8 (–86.5, 32.5) | –46.5 (–265.0, 43.0) | –60.8 (–231.0, 35.0) | –41.5 (1124.0, 43.0) |
| Missing data, n | 2 | 3 | 18 | 0 | 23 |
| Duration of UCD decompensation, days | | | | | |
| Mean (SD) | 7.8 (8.1) | 5.6 (6.5) | 8.7 (12.0) | 16.5 (27.7) | 8.6 (12.1) |
| Median (range) | 6.5 (1.0, 17.0) | 3.0 (1.0, 23.0) | 6.0 (1.0, 82.0) | 3.5 (1.0, 58.0) | 5.0 (1.0, 82.0) |
| Missing data, n | 0 | 0 | 0 | 0 | 0 |
| Duration of periods without decompensation, years* | | | | | |
| N | 2 | 11 | 52 | 2 | 67 |
| Mean (SD) | 2.5 (3.0) | 1.7 (1.5) | 1.8 (2.7) | 4.3 (2.8) | 1.9 (2.6) |
| Median (range) | 2.5 (0.4, 4.6) | 1.1 (0.1, 4.3) | 0.8 (0.0, 12.5) | 4.3 (2.3, 6.3) | 0.9 (0.0, 12.5) |
| Missing data, n | 0 | 0 | 1 | 0 | 1 |

*Duration defined as the time between the response (discharge visit if the episode occurred during a registered decompensation visit or date of the latest ammonium value evaluated at the follow-up visit) and the next visit with documented UCD decompensation.

SD, standard deviation; UCD, urea cycle disorders.

**(b)**

|  | **Time of diagnosis** | |  |
| --- | --- | --- | --- |
|  | **>1 month (N=38)** | **Neo-Natal (N=28)** | **Total (N=66)** |
| Number of Decompensations (diagnosis episode excluded) |  |  |  |
| n | 38 | 28 | 66 |
| 0 | 26 (68.4%) | 10 (35.7%) | 36 (54.5%) |
| 1 | 6 (15.8%) | 4 (14.3%) | 10 (15.2%) |
| 2 | 2 (5.3%) | 5 (17.9%) | 7 (10.6%) |
| >2 | 4 (10.5%) | 9 (32.1%) | 13 (19.7%) |
| Missing data | 0 | 0 | 0 |
| Overall Decompensations Frequency (diagnosis episode excluded, per year) |  |  |  |
| n | 38 | 28 | 66 |
| Mean (SD) | 0.06 (0.12) | 0.76 (1.56) | 0.35 (1.07) |
| Median | 0.00 | 0.19 | 0.00 |
| Q1 - Q3 | 0.00 - 0.05 | 0.00 - 0.51 | 0.00 - 0.21 |
| Range | 0.00 - 0.60 | 0.00 - 5.46 | 0.00 - 5.46 |
| Missing data | 0 | 0 | 0 |
| **Decompensations incidence by treatment, all patients pooled** |  |  |  |
| Global Number of Decompensations per Year under Arginine (diagnosis episode excluded) | 0.03 | 0.08 | 0.04 |
| Global Number of Decompensations per Year under Citrulline (diagnosis episode excluded) | 0.03 | 0.26 | 0.10 |
| Global Number of Decompensations per Year under Arginine and Citrulline (diagnosis episode excluded) | 0.09 | 0.36 | 0.20 |

**Supplementary Table 5.** Evolution of patient development and social life of the exposed population. Data shown are number of patients, and percentages calculated using the number of evaluated patients with available data.

|  | **Patient’s reference visit (n=79)** | **Patient’s last visit  (n=78*)** |
| --- | --- | --- |
| Behavior |  |  |
| Normal, adapted | 69 (100.0) | 75 (98.7) |
| Agitation, major aggressiveness | 0 | 1 (1.3) |
| Not evaluated | 10 | 2 |
| Social life |  |  |
| Normal school / work | 18 (81.8) | 49 (79.0) |
| Adapted school / work | 4 (18.2) | 7 (11.3) |
| No social life / non-independent life | 0 | 6 (9.7) |
| Not evaluated | 57 | 16 |
| Psychomotor development |  |  |
| Normal | 63 (85.1) | 65 (84.4) |
| Impaired | 11 (14.9) | 12 (15.6) |
| Not evaluated | 5 | 1 |

*Data were missing for one patient.

**Supplementary Table 6.** Treatment-related adverse drug reactions in the exposed population.

|  | **Arginine (n=68*)** | | **Citrulline (n=68*)** | | **All patients (n=79)** | |
| --- | --- | --- | --- | --- | --- | --- |
|  | **Patients, n (%)** | **Events, n** | **Patients, n (%)** | **Events, n** | **Patients, n (%)** | **Events, n** |
| At least one ADR | 1 (1.5) | 1 | 1 (1.5) | 2 | 2 (2.5) | 3 |
| Gastrointestinal events | 1 (1.5) | 1 | 1 (1.5) | 2 | 2 (2.5) | 3 |
| Abdominal pain | 0 | 0 | 1 (1.5) | 1 | 1 (1.3) | 1 |
| Nausea | 1 (1.5) | 1 | 1 (1.5) | 1 | 2 (2.5) | 2 |
| ADR leading to treatment discontinuation | 0 | `0 | 1 (1.5) | 2 | 1 (1.3) | 2 |

*Patient numbers are those who received this treatment at least once during follow up.

ADR, adverse drug reaction.

## **Supplementary Table 7.** Overall Outcome during Last Evaluation.

|  | **Time of diagnosis** | | **Diagnosis > 1 month** | | | **Neo-Natal patients** | | | |  |
| --- | --- | --- | --- | --- | --- | --- | --- | --- | --- | --- |
|  | **>1 month (N=48)** | **Neo-Natal (N=31)** | **CPS1 (N=5)** | **HHH (N=2)** | **OTC (N=41)** | **CPS1 (N=10)** | **HHH (N=1)** | **NAGS (N=4)** | **OTC (N=16)** | **Total (N=79)** |
| Overall Outcome during Last Evaluation Visit |  |  |  |  |  |  |  |  |  |  |
| n | 48 | 31 | 5 | 2 | 41 | 10 | 1 | 4 | 16 | 79 |
| Death | 0 (0.0%) | 4 (12.9%) |  |  |  | 1 (10.0%) | 0 (0.0%) | 1 (25.0%) | 2 (12.5%) | 4 (5.1%) |
| Recovered after liver transplantation | 1 (2.1%) | 4 (12.9%) | 0 (0.0%) | 0 (0.0%) | 1 (2.4%) | 1 (10.0%) | 0 (0.0%) | 0 (0.0%) | 3 (18.8%) | 5 (6.3%) |
| Regular general state (balanced treatment, regular daily life) | 47 (97.9%) | 22 (71.0%) | 5 (100%) | 2 (100%) | 40 (97.6%) | 8 (80.0%) | 1 (100%) | 3 (75.0%) | 10 (62.5%) | 69 (87.3%) |
| Unbalanced state (frequent consultation or hospitalization, unbalanced treatment) | 0 (0.0%) | 1 (3.2%) |  |  |  | 0 (0.0%) | 0 (0.0%) | 0 (0.0%) | 1 (6.3%) | 1 (1.3%) |
| Missing data | 0 | 0 | 0 | 0 | 0 | 0 | 0 | 0 | 0 | 0 |
| Treatment Group the Day Before Liver Transplantation |  |  |  |  |  |  |  |  |  |  |
| n | 1 | 4 |  |  | 1 | 1 |  |  | 3 | 5 |
| Arginine and Citrulline | 1 (100%) | 4 (100%) |  |  | 1 (100%) | 1 (100%) |  |  | 3 (100%) | 5 (100%) |
| Missing data | 0 | 0 |  |  | 0 | 0 |  |  | 0 | 0 |
