# Supplementary material for: Citrulline in the management of patients with urea cycle disorders
Source: Orphanet J Rare Dis. 2023 Jul 21;18:207. doi: 10.1186/s13023-023-02800-8 (PMC10362745; doi:10.1186/s13023-023-02800-8)
Supplement: Supplementary file 1 — Supplementary Material 1 [file 13023_2023_2800_MOESM1_ESM.pdf]

DocuSign Envelope ID: 062F7A96-90A2-4AC9-A17F-9519171B35B7

Catherine Rees
Independent Medical Writer and Editor
12 Nicholson Place, Hillcrest | Auckland 0627 | New Zealand
Email: catherinejrees@gmail.com

21 October 2022

Dear Editor,

Re: Citrulline in the management of patients with urea cycle disorders

I confirm that editorial assistance was provided by myself, Catherine Rees, on behalf of Springer Healthcare Communications, and that this support was funded by Biocodex. I give permission for these details to be included in the Acknowledgements section should the manuscript be accepted for publication by *Orphanet Journal of Rare Diseases.*

Yours sincerely,

DocuSigned by:
EA09D2C0B963416...

Catherine Rees
On behalf of Springer Healthcare Communications
